# Supplementary material for: Unlocking the return insurance puzzle in e-commerce: A strategic dance between e-sellers and the e-platform
Source: PLoS One. 2025 May 19;20(5):e0322376. doi: 10.1371/journal.pone.0322376 (PMC12088037; doi:10.1371/journal.pone.0322376)
Supplement: S1 Appendix — (DOCX) [file pone.0322376.s001.docx]

**Supplementary Materials**

**Appendix A- Proof of Proposition**

**The following three proofs are used to demonstrate that Propositions 1-3 hold true.**

Proof of Proposition 1:

$p_{2}^{NO*}-p_{2}^{NN*}=\frac{4\alpha_{1}f_{2}-\left[2+\left(\alpha_{2}-1\right)h\right]\left(2\alpha_{1}-\alpha_{2}\right)h\left(\alpha_{2}-1\right)\left(1-\beta\right)}{2\left(4\alpha_{1}-\alpha_{2}\right)\left(1-\beta\right)}>0$.$p_{1}^{ON*}-p_{1}^{NN*}=\frac{4\alpha_{1}f_{2}+\left[2+\left(\alpha_{1}-1\right)h\right]h\left(1-\alpha_{1}\right)\left(1-\beta\right)\left(2\alpha_{1}-\alpha_{2}\right)}{2\left(4\alpha_{1}-\alpha_{2}\right)\left(1-\beta\right)\alpha_{1}}>0$. $D_{2}^{NO*}-D_{2}^{NN*}=-\frac{\left\{2f_{2}+\left[2+\left(\alpha_{2}-1\right)h\right]h\left(\alpha_{2}-1\right)\left(1-\beta\right)\right\}\alpha_{1}\left(2\alpha_{1}-\alpha_{2}\right)}{2\left(4\alpha_{1}-\alpha_{2}\right)\left(\alpha_{1}-\alpha_{2}\right)\left(1-\beta\right)\alpha_{2}}$, $p_{1}^{NO*}-p_{1}^{NN*}=\frac{2f_{2}+\left[2+\left(\alpha_{2}-1\right)h\right]h\left(\alpha_{2}-1\right)\left(1-\beta\right)}{2\left(4\alpha_{1}-\alpha_{2}\right)\left(1-\beta\right)}$, $D_{1}^{NO*}-D_{1}^{NN*}=\frac{\left\{2f_{2}+\left[2+\left(\alpha_{2}-1\right)h\right]h\left(\alpha_{2}-1\right)\left(1-\beta\right)\right\}\alpha_{1}}{2\left(4\alpha_{1}-\alpha_{2}\right)\left(\alpha_{1}-\alpha_{2}\right)\left(1-\beta\right)}$, so when $f_{2}>\frac{\left[2+\left(\alpha_{2}-1\right)h\right]h\left(1-\alpha_{2}\right)\left(1-\beta\right)}{2}$, $D_{2}^{NO*}-D_{2}^{NN*}<0$, $p_{1}^{NO*}-p_{1}^{NN*}>0$, $D_{1}^{NO*}-D_{1}^{NN*}>0$. $D_{1}^{ON*}-D_{1}^{NN*}=-\frac{\left(2\alpha_{1}-\alpha_{2}\right)\left\{2f_{1}-\left[2+\left(\alpha_{1}-1\right)h\right]h\left(1-\alpha_{1}\right)\left(1-\beta\right)\right\}}{2\left(4\alpha_{1}-\alpha_{2}\right)\left(\alpha_{1}-\alpha_{2}\right)\left(1-\beta\right)}$, $p_{2}^{ON*}-p_{2}^{NN*}=\frac{2f_{1}-\left[2+\left(\alpha_{1}-1\right)h\right]h\left(1-\alpha_{1}\right)\left(1-\beta\right)}{2\left(4\alpha_{1}-\alpha_{2}\right)\left(1-\beta\right)}$, $D_{2}^{ON*}-D_{2}^{NN*}=\frac{\left\{2f_{1}-\left[2+\left(\alpha_{1}-1\right)h\right]h\left(1-\alpha_{1}\right)\left(1-\beta\right)\right\}\alpha_{1}}{2\left(4\alpha_{1}-\alpha_{2}\right)\left(\alpha_{1}-\alpha_{2}\right)\left(1-\beta\right)}$, so when $f_{1}<\frac{\left[2+\left(\alpha_{1}-1\right)h\right]h\left(1-\alpha_{1}\right)\left(1-\beta\right)}{2}$, $D_{1}^{ON*}-D_{1}^{NN*}>0$, $p_{2}^{ON*}-p_{2}^{NN*}<0$, $D_{2}^{ON*}-D_{2}^{NN*}<0$. $\pi_{1}^{NO*}-\pi_{1}^{NO*}=\left(1-\beta\right)\left(\alpha_{1}-\alpha_{2}\right)\left(D_{1}^{NO*}-D_{1}^{NN*}\right)\left(D_{1}^{NO*}+D_{1}^{NN*}\right)$, $\pi_{2}^{NO*}-\pi_{2}^{NN*}=\frac{\left(1-\beta\right)\alpha_{2}\left(\alpha_{1}-\alpha_{2}\right)\left(D_{2}^{NO*}-D_{2}^{NN*}\right)\left(D_{2}^{NO*}+D_{2}^{NN*}\right)}{\alpha_{1}}$. $\pi_{1}^{ON*}-\pi_{1}^{NN*}=\left(1-\beta\right)\left(\alpha_{1}-\alpha_{2}\right)\left(D_{1}^{ON*}-D_{1}^{NN*}\right)\left(D_{1}^{ON*}+D_{1}^{NN*}\right)$, $\pi_{2}^{ON*}-\pi_{2}^{NN*}=\frac{\left(1-\beta\right)\alpha_{2}\left(\alpha_{1}-\alpha_{2}\right)\left(D_{2}^{ON*}-D_{2}^{NN*}\right)\left(D_{2}^{ON*}+D_{2}^{NN*}\right)}{\alpha_{1}}$.

Proof of Proposition 2:

Using the crossing method, it becomes evident that when $\pi_{2}^{NO*}<\pi_{2}^{NN*}$ and $\pi_{1}^{ON*}<\pi_{1}^{NN*}$, not providing return insurance emerges as the equilibrium strategy. Referring to the Proof of Proposition 1, in this particular scenario, it can be deduced that $f_{1}>\frac{\left[2+\left(\alpha_{1}-1\right)h\right]h\left(1-\alpha_{1}\right)\left(1-\beta\right)}{2}$, and $f_{2}>\frac{\left[2+\left(\alpha_{2}-1\right)h\right]h\left(1-\alpha_{2}\right)\left(1-\beta\right)}{2}$. When $\pi_{2}^{ON*}>\pi_{2}^{OO*}$ and $\pi_{1}^{ON*}>\pi_{1}^{NN*}$, the equilibrium strategy is for only high-quality e-tailer to offer return insurance. Since $D_{2}^{ON*}-D_{2}^{OO*}=\frac{\left\{2f_{2}-\left[2+\left(\alpha_{1}-1\right)h\right]h\left(1-\alpha_{2}\right)\left(1-\beta\right)\right\}\alpha_{1}}{2\left(4\alpha_{1}-\alpha_{2}\right)\left(\alpha_{1}-\alpha_{2}\right)\left(1-\beta\right)}$ and $\pi_{2}^{ON*}-\pi_{2}^{OO*}=\frac{\left(1-\beta\right)\alpha_{2}\left(\alpha_{1}-\alpha_{2}\right)\left(D_{2}^{ON*}-D_{2}^{OO*}\right)\left(D_{2}^{ON*}+D_{2}^{OO*}\right)}{\alpha_{1}}$, it follows that when $f_{2}>\frac{\left[2+\left(\alpha_{2}-1\right)h\right]h\left(1-\alpha_{2}\right)\left(1-\beta\right)}{2}$ , $\pi_{2}^{ON*}>\pi_{2}^{OO*}$. In combination with the Proof of Proposition 1, we observe that $f_{1}<\frac{\left[2+\left(\alpha_{1}-1\right)h\right]h\left(1-\alpha_{1}\right)\left(1-\beta\right)}{2}$and $f_{2}>\frac{\left[2+\left(\alpha_{2}-1\right)h\right]h\left(1-\alpha_{2}\right)\left(1-\beta\right)}{2}$. When $\pi_{2}^{NO*}>\pi_{2}^{NN*}$ and $\pi_{1}^{NO*}>\pi_{1}^{OO*}$, only low-quality e-seller offer return insurance as the equilibrium strategy. Because $D_{1}^{NO*}-D_{1}^{OO*}=\frac{\left(2\alpha_{1}-\alpha_{2}\right)\left\{2f_{1}-\left[2+\left(\alpha_{1}-1\right)h\right]h\left(1-\alpha_{1}\right)\left(1-\beta\right)\right\}}{2\left(4\alpha_{1}-\alpha_{2}\right)\left(\alpha_{1}-\alpha_{2}\right)\left(1-\beta\right)}$, $\pi_{1}^{NO*}-\pi_{1}^{OO*}=\left(1-\beta\right)\left(\alpha_{1}-\alpha_{2}\right)\left(D_{1}^{NO*}-D_{1}^{OO*}\right)\left(D_{1}^{NO*}+D_{1}^{OO*}\right)$, so when $f_{1}>\frac{\left[2+\left(\alpha_{1}-1\right)h\right]h\left(1-\alpha_{1}\right)\left(1-\beta\right)}{2}$, $\pi_{1}^{NO*}>\pi_{1}^{OO*}$. Combined with Proof of Proposition 1, $f_{1}>\frac{\left[2+\left(\alpha_{1}-1\right)h\right]h\left(1-\alpha_{1}\right)\left(1-\beta\right)}{2}$, $f_{2}<\frac{\left[2+\left(\alpha_{2}-1\right)h\right]h\left(1-\alpha_{2}\right)\left(1-\beta\right)}{2}$. When $\pi_{2}^{OO*}>\pi_{2}^{ON*}$ and $\pi_{1}^{NO*}<\pi_{1}^{OO*}$, both e-sellers offer return insurance as a balancing strategy. In this scenario, $f_{1}<\frac{\left[2+\left(\alpha_{1}-1\right)h\right]h\left(1-\alpha_{1}\right)\left(1-\beta\right)}{2}$, and $f_{2}<\frac{\left[2+\left(\alpha_{2}-1\right)h\right]h\left(1-\alpha_{2}\right)\left(1-\beta\right)}{2}$.

Proof of Proposition 3:

$\mathop{p_{2}^{NO*}}\limits^{\sim} -\mathop{p_{2}^{NN*}}\limits^{\sim} =\frac{2\alpha_{1}f_{2}}{\alpha_{2}\left(4\alpha_{1}-\alpha_{2}\right)\left(1-\beta\right)}>0$, $\mathop{p_{1}^{ON*}}\limits^{\sim} -\mathop{p_{1}^{NN*}}\limits^{\sim} =\frac{2f_{2}}{\left(4\alpha_{1}-\alpha_{2}\right)\left(1-\beta\right)}>0$. $\mathop{D_{2}^{NO*}}\limits^{\sim} -\mathop{D_{2}^{NN*}}\limits^{\sim} =-\frac{\alpha_{1}f_{2}\left(2\alpha_{1}-\alpha_{2}\right)}{\left(4\alpha_{1}-\alpha_{2}\right)\left(\alpha_{1}-\alpha_{2}\right)\left(1-\beta\right)\alpha_{2}}<0$, $\mathop{p_{1}^{NO*}}\limits^{\sim} -\mathop{p_{1}^{NN*}}\limits^{\sim} =\frac{f_{2}}{\left(4\alpha_{1}-\alpha_{2}\right)\left(1-\beta\right)}>0$, $\mathop{D_{1}^{NO*}}\limits^{\sim} -\mathop{D_{1}^{NN*}}\limits^{\sim} =\frac{\alpha_{1}f_{2}}{\left(4\alpha_{1}-\alpha_{2}\right)\left(\alpha_{1}-\alpha_{2}\right)\left(1-\beta\right)}>0$. $\mathop{D_{1}^{ON*}}\limits^{\sim} -\mathop{D_{1}^{NN*}}\limits^{\sim} =-\frac{\left(2\alpha_{1}-\alpha_{2}\right)f_{1}}{\left(4\alpha_{1}-\alpha_{2}\right)\left(\alpha_{1}-\alpha_{2}\right)\left(1-\beta\right)}<0$, $\mathop{p_{2}^{ON*}}\limits^{\sim} -\mathop{p_{2}^{NN*}}\limits^{\sim} =\frac{f_{1}}{\left(4\alpha_{1}-\alpha_{2}\right)\left(1-\beta\right)}>0$, $\mathop{D_{2}^{ON*}}\limits^{\sim} -\mathop{D_{2}^{NN*}}\limits^{\sim} =\frac{\alpha_{1}f_{1}}{\left(4\alpha_{1}-\alpha_{2}\right)\left(\alpha_{1}-\alpha_{2}\right)\left(1-\beta\right)}>0$. $\mathop{\pi_{1}^{NO*}}\limits^{\sim} -\mathop{\pi_{1}^{NN*}}\limits^{\sim} =\left(1-\beta\right)\left(\alpha_{1}-\alpha_{2}\right)\left(\mathop{D_{1}^{NO*}}\limits^{\sim} -\mathop{D_{1}^{NN*}}\limits^{\sim} \right)\left(\mathop{D_{1}^{NO*}}\limits^{\sim} +\mathop{D_{1}^{NN*}}\limits^{\sim} \right)$, $\mathop{\pi_{2}^{NO*}}\limits^{\sim} -\mathop{\pi_{2}^{NN*}}\limits^{\sim} =\frac{\left(1-\beta\right)\alpha_{2}\left(\alpha_{1}-\alpha_{2}\right)\left(\mathop{D_{2}^{NO*}}\limits^{\sim} -\mathop{D_{2}^{NN*}}\limits^{\sim} \right)\left(\mathop{D_{2}^{NO*}}\limits^{\sim} +\mathop{D_{2}^{NN*}}\limits^{\sim} \right)}{\alpha_{1}}$. $\mathop{\pi_{1}^{ON*}}\limits^{\sim} -\mathop{\pi_{1}^{NN*}}\limits^{\sim} =\left(1-\beta\right)\left(\alpha_{1}-\alpha_{2}\right)\left(\mathop{D_{1}^{ON*}}\limits^{\sim} -\mathop{D_{1}^{NN*}}\limits^{\sim} \right)\left(\mathop{D_{1}^{ON*}}\limits^{\sim} +\mathop{D_{1}^{NN*}}\limits^{\sim} \right)$, $\mathop{\pi_{2}^{ON*}}\limits^{\sim} -\mathop{\pi_{2}^{NN*}}\limits^{\sim} =\frac{\left(1-\beta\right)\alpha_{2}\left(\alpha_{1}-\alpha_{2}\right)\left(\mathop{D_{2}^{ON*}}\limits^{\sim} -\mathop{D_{2}^{NN*}}\limits^{\sim} \right)\left(\mathop{D_{2}^{ON*}}\limits^{\sim} +\mathop{D_{2}^{NN*}}\limits^{\sim} \right)}{\alpha_{1}}$.
