# Supplementary material for: Unlocking the return insurance puzzle in e-commerce: A strategic dance between e-sellers and the e-platform
Source: PLoS One. 2025 May 19;20(5):e0322376. doi: 10.1371/journal.pone.0322376 (PMC12088037; doi:10.1371/journal.pone.0322376)
Supplement: Attachment — Submitted filename: Response to Reviewers.docx [file pone.0322376.s003.docx]

Dear Ayatulloh Michael Musyaffi,

Thank you very much for giving us an opportunity to revise our manuscript. We appreciate the editor and reviewers very much for their constructive comments and suggestions on our manuscript entitled ***“Unlocking the Return Insurance Puzzle in E-Commerce: A Strategic Dance Between E-Sellers and E-Platforms”***(PONE-D-24-22624). Those comments are all valuable and very helpful for revising and improving our paper, as well as the important guiding significance to our researches. We have studied comments carefully and have made correction which we hope meet with approval.

First, we will reply to the editor's suggestions one by one.

**1. Please ensure that your manuscript meets PLOS ONE's style requirements, including those for file naming. The PLOS ONE style templates can be found at**

**https://journals.plos.org/plosone/s/file?id=wjVg/PLOSOne_formatting_sample_main_body.pdf and**

[**https://journals.plos.org/plosone/s/file?id=ba62/PLOSOne_formatting_sample_title_authors_affiliations.pdf**](https://journals.plos.org/plosone/s/file?id=ba62/PLOSOne_formatting_sample_title_authors_affiliations.pdf)

**The author’s answer:** Dear Editor, thank you very much for your advice! We have revised the format of the paper according to the requirements in the link.

**2. Thank you for stating the following financial disclosure: "Postgraduate Innovative Research Projects of Hainan Province(Qhyb2022-20 and Qhyb2021-19)" 
Please state what role the funders took in the study.  If the funders had no role, please state: ""The funders had no role in study design, data collection and analysis, decision to publish, or preparation of the manuscript."" 
If this statement is not correct you must amend it as needed. 
Please include this amended Role of Funder statement in your cover letter; we will change the online submission form on your behalf.**

**The author’s answer:** Dear Editor, thank you very much for your carefulness. After discussion, we have removed the funding with the fund number "Qhyb2021-19". The revised financial disclosure is "Postgraduate Innovative Research Projects of Hainan Province (Qhyb2022-20)". The funder, Zhang Chen, has role in the study design, data collection and analysis, decision to publish, and preparation of the manuscript. We have added the financial disclosure and the role of the funder in the research to the cover letter and highlighted it in yellow. Please refer to the re-uploaded cover letter for details.

**3. We note that your Data Availability Statement is currently as follows: [All relevant data are within the manuscript and its Supporting Information files]**

**Please confirm at this time whether or not your submission contains all raw data required to replicate the results of your study. Authors must share the “minimal data set” for their submission. PLOS defines the minimal data set to consist of the data required to replicate all study findings reported in the article, as well as related metadata and methods (**[**https://journals.plos.org/plosone/s/data-availability#loc-minimal-data-set-definition**](https://journals.plos.org/plosone/s/data-availability#loc-minimal-data-set-definition)**).**

**For example, authors should submit the following data:**

**- The values behind the means, standard deviations and other measures reported;
- The values used to build graphs;
- The points extracted from images for analysis.**

**Authors do not need to submit their entire data set if only a portion of the data was used in the reported study.**

**If your submission does not contain these data, please either upload them as Supporting Information files or deposit them to a stable, public repository and provide us with the relevant URLs, DOIs, or accession numbers. For a list of recommended repositories, please see** [**https://journals.plos.org/plosone/s/recommended-repositories**](https://journals.plos.org/plosone/s/recommended-repositories)**.**

**If there are ethical or legal restrictions on sharing a de-identified data set, please explain them in detail (e.g., data contain potentially sensitive information, data are owned by a third-party organization, etc.) and who has imposed them (e.g., an ethics committee). Please also provide contact information for a data access committee, ethics committee, or other institutional body to which data requests may be sent. If data are owned by a third party, please indicate how others may request data access.**

**The author’s answer:** Dear Editor, thank you very much for your carefulness. Upon reconfirmation, the values used to construct all the figures are already included in our manuscript.

Next, the main corrections in the paper and the responds to the reviewer’s comments are as following:

**Reviewer #1:**

**In my opinion, overall, the abstract accurately reflects the content of the research article. The Introduction section also clearly states the research problem in the form of a question. However, it would be better if gaps from previous studies on the same topic were included, so that the gaps are not only practical but also from previous research.**

**The author’s answer:** Dear Reviewer, thank you very much for your recognition and valuable suggestions! Based on your advice, we have added in the introduction section the differences between our manuscript and previous studies on the same topic, and we have used the practical example of T-mall offering return insurance to consumers every month to demonstrate the rationality of our manuscript. The specific revisions are as follows: (please refer to the highlighted paragraph at the end of page 3 in the revised manuscript for details)

“Unfortunately, there are still some gaps in the current research on return insurance. Most of the research on return insurance focuses on a single-channel environment [1-5]. Research on return insurance in a competitive environment mainly explores the strategic choices of competitive e-sellers, rarely considering the impact of commission rates on the strategic choices of e-sellers' return insurance. [6, 7] No research has taken e-platforms as the game subject to explore the optimal return policy of e-platforms. The research on the online return policy of e-platforms and its impact on e-sellers is still an unresolved issue. In fact, some e-platforms improve the return service by presenting consumers with equity version of return insurance, shipping insurance cards, etc. in order to attract them to buy. For example, T-mall gives away return insurance to consumers every month based on their consumption amount and other information on the platform. Therefore, it is necessary to explore the return insurance strategy of e-platforms and its impact on the strategies of e-sellers.”

**In the literature review section, several sources have been included, although there is still room to add more sources to increase the number of references.**

**The author’s answer:** Dear Reviewer, thank you very much for your suggestions! In accordance with your suggestions, we have added highly relevant literature to our manuscript. The newly added several sources are as follows (please refer to the highlighted section in the "References" part of the revised manuscript for details):

“1.	Li XX, Gao J, Bian YW. Return freight insurance strategies for the online retailer and insurance company. International Journal of Production Economics. 2023; 256:108752. <https://doi.org/10.1016/j.ijpe.2022.108752>

6.	Xiong H, Liu J. Return insurance in a competitive market: Benefiting the high-quality or low-quality retailer?. International Journal of Production Economics. 2023; 255:108719. <https://doi.org/10.1016/j.ijpe.2022.108719>

9.	Geng SD, Li WL. Complimentary Return-Freight Insurance Serves as Quality Signal or Noise?. ACIS 2017 Proceedings. 2017; 1-10. https://aisel.aisnet.org/acis2017/47

10.	Lin JX, Choi TM, Kuo YH. Will Providing Return-Freight-Insurances Do More Good than Harm to Dual-Channel E-Commerce Retailers?. European Journal of Operational Research. 2023; 307(3):1225-1239. https://doi.org/10.1016/j.ejor.2022.09.025

11.	Zhao XM, Hu SH, Meng XX. Who should pay for return freight in the online retailing? Retailers or consumers. Electronic Commerce Research. 2020; 20(2):427-452. https://link.springer.com/article/10.1007/s10660-019-09360-9

12.	Zhang TL, Guo XF, Wu T. An analysis of cross-channel return processing with return-freight insurance for live streaming platforms. Computers & Industrial Engineering. 2022; 174:108805. <https://doi.org/10.1016/j.cie.2022.108805>

18.	Das L, Kunja SR. Why do consumers return products? A qualitative exploration of online product return behaviour of young consumers. Journal of Retailing and Consumer Services. 2024; 78:103770. <https://doi.org/10.1016/j.jretconser.2024.103770>

21.	Wang C, Chen J, Chen X. Pricing and order decisions with option contracts in the presence of customer returns. International Journal of Production Economics. 2017; 193:422-436. <https://doi.org/10.1016/j.ijpe.2017.08.011>

23.	Liu JQ, Yuan R, Feng S. Whether to offer return-freight insurance or open a physical showroom: A strategic analysis of an online retailer’s decisions. Journal of Retailing and Consumer Services. 2023; 75:103498. https://doi.org/10.1016/j.jretconser.2023.103498

24.	Jin DL, Huang M. Competing e-tailers’ adoption strategies of buy-online-and-return-in-store service. Electronic Commerce Research and Applications. 2021; 47:101047. https://doi.org/10.1016/j.elerap.2021.101047

25.	Yang L, Li XY, Xia Y, Aneja YP. Returns operations in omnichannel retailing with buy-online-and-return-to-store. Omega. 2023; 119:102874. https://doi.org/10.1016/j.omega.2023.102874

26.	Huang M, Jin DL. Impact of buy-online-and-return-in-store service on omnichannel retailing: A supply chain competitive perspective. Electronic Commerce Research and Applications. 2020; 41:100977. https://doi.org/10.1016/j.elerap.2020.100977

27.	Yan S, Archibald TW, Han XH, Bian YW. Whether to adopt “buy online and return to store” strategy in a competitive market?. European Journal of Operational Research. 2022; 301(3):974-986. <https://doi.org/10.1016/j.ejor.2021.11.040>”

In addition, in order to better highlight the newly added literature, a description of the new literature has been added to the literature review section (please refer to the "Literature Review" section in the revised manuscript, highlighted in yellow):

1) The "Return Insurance" section has been enriched with descriptions of the research conducted by scholars such as Lin and Zhao:

“For example, Lin et al. explore whether dual-channel retailers should offer return freight insurance and find that dual-channel retailers should offer return freight insurance when the production cost, salvage value, and return freight insurance cost of the goods are all high [10]. Zhao and Hu find that it is more effective for retailers to give away return freight insurance from profit and social welfare perspectives [11].”

2) The "Consumer Return" section has been augmented with descriptions of the research conducted by scholars such as Xu, Yan, and Wang.

“For example, Xu et al. believed that under the return policy, consumers' valuation of goods depends on the return amount and the length of time they have to wait after returning the goods, and systematically investigated the impact of the return deadline on consumer behavior[14]. In addition, some scholars have also studied the management decisions related to consumer returns[20-23]. For example, Yan et al. [2] regarded consumer returns as private information and studied the value of product return information to supply chain companies[20]. Wang et al. (2017) [9] studied the pricing, ordering, and return policies of option contracts for retailers with customer returns under stochastic demand[21].”

**The methods and results sections have been explained in detail.**

**The author’s answer:** Dear Reviewer, thank you very much for your recognition!

**In the conclusion section, add other implications that might be beneficial for stakeholders and policymakers.**

**The author’s answer:** Dear Reviewer, thank you very much for your suggestions! Based on your comments, additional implications that may be beneficial to stakeholders and policymakers have been included in the conclusion section (please refer to page 29 of the revised manuscript, highlighted in yellow).

1) Based on the previous two conclusions regarding e-sellers' pricing strategies and return insurance policies, this revision proposes suggestions focused on formulating e-sellers' pricing and return insurance policies:

“Firstly, based on the conditions for e-sellers to offer return insurance, we recommend that e-sellers should comprehensively consider factors such as the product return rate, return compensation, and the commission rate of e-platforms from the perspectives of themselves, insurance companies, and e- platforms to determine the size of the insurance premium before selecting a return strategy. If providing return insurance is not the most suitable return strategy, e-sellers can reduce product prices to attract consumers to purchase by offering discounts and induce consumers to purchase return shipping insurance on their own to reduce return losses.”

2) Based on the final conclusion regarding e-platforms' return insurance policies, this revision proposes suggestions focused on e-platforms:

“Secondly, based on the optimal return insurance strategy of e-platforms and its impact on e-sellers, we suggest that e-platforms should play their due role by taking certain measures to increase consumer loyalty without interfering with the market, and should not proactively offer return shipping insurance to consumers.”

**Reviewer #2: This article establishes a duopoly game model to find the best return insurance policy and its impact on e-sellers and e-platforms. In addition, the following are some suggestions that can be included in the revision.**

**The author’s answer:** Dear Reviewer, thank you very much for your comments! Next, we will respond to each of your suggestions point by point.

**(1) The language is colloquial and the English level of the paper needs to be improved.**

**The author’s answer:** Dear Reviewer, thank you very much for your suggestions! Based on your comments, we sought the help of scholars who have published papers in SCI journals and have revised and polished the entire manuscript. Given that the polishing was extensive and involved numerous revised sentences throughout the text, in order to highlight other more critical suggestions, we have not specifically marked the polished sentences. Below, I will illustrate the changes made to the last paragraph of the Introduction before and after the revision.

Before polishing:

“The remainder of this study is organized as follows. Part 2 reviews pertinent literature, while Part 3 provides a detailed description of the model. Part 4 calculates the optimal pricing strategy, demand, and profit for e-sellers of different qualities without free return insurance on the e-platform. This section also analyzes the return insurance policy choices of high-quality and low-quality e-sellers in a competitive environment. Part 5 extends this analysis to scenarios where the e-platform provides return insurance, examining optimal pricing strategies, demand, profit, and return insurance policies for e-sellers with varying qualities. The section also explores the influence of return insurance provided by the e-platform on the profit of e-sellers with different qualities and the optimal return insurance policy for the e-platform. Finally, Part 6 concludes the study.”

After polishing:

“The structure of this study is organized as follows. Section 2 offers a summary of relevant literature. Section 3 details the introduction of the model. Section 4 calculates the optimal pricing strategy, demand, and profit for e-sellers of different qualities without free return insurance on the e-platform. This section also analyzes the return insurance policy choices of high-quality and low-quality e-sellers in a competitive environment. Section 5 extends this analysis to scenarios where the e-platform offers return insurance, examining optimal pricing strategies, demand, profit, and return insurance policies for e-sellers with varying qualities. The section also explores the influence of return insurance offered by the e-platform on the profit of e-sellers with different qualities and the optimal return insurance policy for the e-platform. Section 6 presents the conclusions.”

**(2) The introduction section can briefly introduce the current research and hypotheses on return insurance policies.**

**The author’s answer:** Dear Reviewer, thank you very much for your suggestions! Based on your suggestions, we have revised the “Introduction” section to include the current research status and hypotheses regarding return insurance policies, as well as to highlight the differences between our manuscript and previous related literature. Specifically, the modifications are as follows: (Please refer to the last paragraph on page 3 of the revised manuscript, which is highlighted in yellow)

“Unfortunately, there are still some gaps in the current research on return insurance. Most of the research on return insurance focuses on a single-channel environment [1-5]. Research on return insurance in a competitive environment mainly explores the strategic choices of competitive e-sellers, rarely considering the impact of commission rates on the strategic choices of e-sellers' return insurance. [6, 7] No research has taken e-platforms as the game subject to explore the optimal return policy of e-platforms. The research on the online return policy of e-platforms and its impact on e-sellers is still an unresolved issue. In fact, some e-platforms improve the return service by presenting consumers with equity version of return insurance, shipping insurance cards, etc. in order to attract them to buy. For example, T-mall gives away return insurance to consumers every month based on their consumption amount and other information on the platform. Therefore, it is necessary to explore the return insurance strategy of e-platforms and its impact on the strategies of e-sellers.”

**(3) The literature review can be appropriately expanded.**

**The author’s answer:** Dear Reviewer, thank you very much for your suggestions! In accordance with your suggestions, we have added highly relevant literature to our manuscript. The newly added several sources are as follows (please refer to the highlighted section in the "References" part of the revised manuscript for details):

“1.	Li XX, Gao J, Bian YW. Return freight insurance strategies for the online retailer and insurance company. International Journal of Production Economics. 2023; 256:108752. <https://doi.org/10.1016/j.ijpe.2022.108752>

6.	Xiong H, Liu J. Return insurance in a competitive market: Benefiting the high-quality or low-quality retailer?. International Journal of Production Economics. 2023; 255:108719. <https://doi.org/10.1016/j.ijpe.2022.108719>

9.	Geng SD, Li WL. Complimentary Return-Freight Insurance Serves as Quality Signal or Noise?. ACIS 2017 Proceedings. 2017; 1-10. https://aisel.aisnet.org/acis2017/47

10.	Lin JX, Choi TM, Kuo YH. Will Providing Return-Freight-Insurances Do More Good than Harm to Dual-Channel E-Commerce Retailers?. European Journal of Operational Research. 2023; 307(3):1225-1239. https://doi.org/10.1016/j.ejor.2022.09.025

11.	Zhao XM, Hu SH, Meng XX. Who should pay for return freight in the online retailing? Retailers or consumers. Electronic Commerce Research. 2020; 20(2):427-452. https://link.springer.com/article/10.1007/s10660-019-09360-9

12.	Zhang TL, Guo XF, Wu T. An analysis of cross-channel return processing with return-freight insurance for live streaming platforms. Computers & Industrial Engineering. 2022; 174:108805. <https://doi.org/10.1016/j.cie.2022.108805>

18.	Das L, Kunja SR. Why do consumers return products? A qualitative exploration of online product return behaviour of young consumers. Journal of Retailing and Consumer Services. 2024; 78:103770. <https://doi.org/10.1016/j.jretconser.2024.103770>

21.	Wang C, Chen J, Chen X. Pricing and order decisions with option contracts in the presence of customer returns. International Journal of Production Economics. 2017; 193:422-436. <https://doi.org/10.1016/j.ijpe.2017.08.011>

23.	Liu JQ, Yuan R, Feng S. Whether to offer return-freight insurance or open a physical showroom: A strategic analysis of an online retailer’s decisions. Journal of Retailing and Consumer Services. 2023; 75:103498. https://doi.org/10.1016/j.jretconser.2023.103498

24.	Jin DL, Huang M. Competing e-tailers’ adoption strategies of buy-online-and-return-in-store service. Electronic Commerce Research and Applications. 2021; 47:101047. https://doi.org/10.1016/j.elerap.2021.101047

25.	Yang L, Li XY, Xia Y, Aneja YP. Returns operations in omnichannel retailing with buy-online-and-return-to-store. Omega. 2023; 119:102874. https://doi.org/10.1016/j.omega.2023.102874

26.	Huang M, Jin DL. Impact of buy-online-and-return-in-store service on omnichannel retailing: A supply chain competitive perspective. Electronic Commerce Research and Applications. 2020; 41:100977. https://doi.org/10.1016/j.elerap.2020.100977

27.	Yan S, Archibald TW, Han XH, Bian YW. Whether to adopt “buy online and return to store” strategy in a competitive market?. European Journal of Operational Research. 2022; 301(3):974-986. <https://doi.org/10.1016/j.ejor.2021.11.040>”

In addition, in order to better highlight the newly added literature, a description of the new literature has been added to the literature review section (please refer to the "Literature Review" section in the revised manuscript, highlighted in yellow):

1) The "Return Insurance" section has been enriched with descriptions of the research conducted by scholars such as Lin and Zhao:

“For example, Lin et al. explore whether dual-channel retailers should offer return freight insurance and find that dual-channel retailers should offer return freight insurance when the production cost, salvage value, and return freight insurance cost of the goods are all high [10]. Zhao and Hu find that it is more effective for retailers to give away return freight insurance from profit and social welfare perspectives [11].”

2) The "Consumer Return" section has been augmented with descriptions of the research conducted by scholars such as Xu, Yan, and Wang.

“For example, Xu et al. believed that under the return policy, consumers' valuation of goods depends on the return amount and the length of time they have to wait after returning the goods, and systematically investigated the impact of the return deadline on consumer behavior[14]. In addition, some scholars have also studied the management decisions related to consumer returns[20-23]. For example, Yan et al. [2] regarded consumer returns as private information and studied the value of product return information to supply chain companies[20]. Wang et al. (2017) [9] studied the pricing, ordering, and return policies of option contracts for retailers with customer returns under stochastic demand[21].”

**(4) The format of the paper is not standardized.**

**The author’s answer:** Dear Reviewer, thank you very much for your suggestions! Based on your suggestions, we have revised the formatting of the paper to align with the template requirements of PLOS ONE.

**(5) Some real business cases can be introduced in the analysis and comparison.**

**The author’s answer:** Dear Reviewer, thank you very much for your advice! According to your suggestions, this revision introduces some real business cases in the analysis and comparison (please refer to the highlighted part in the "Comparative analysis" of the revised manuscript).

1) In the first paragraph of the explanation for Proposition 2, this revision adds the cases of the official flagship store of Suning Tesco and Xiaomi's official flagship store on Taobao platform to demonstrate the rationality of the conclusion that "e-sellers can induce consumers to purchase return insurance on their own at a profit by lowering prices and offering poor return services." Specific changes are as follows: (please refer to the highlighted part in the first paragraph of the explanation for Proposition 2 on page 20 of the revised manuscript)

“According to a search, on the Taobao platform, the official flagship store of Suning Tesco sells a mouse identical to the one sold by Xiaomi's official flagship store, but at a price 21.2 yuan lower than Xiaomi's. Unlike Xiaomi, however, Suning Tesco does not offer consumer return insurance. This further demonstrates the accuracy of this conclusion.”

2) This revision includes additional cases of Estée Lauder and Maybelline, as well as The North Face and Hailan Home, in the second paragraph of the explanation for Proposition 2. These cases are used to support the conclusion that e-sellers' choice of return insurance policy is not influenced by their competitors' policy. Specific changes are as follows: (please refer to the highlighted part in the highlighted section in the second paragraph of the explanation for Proposition 2 on page 20 of the revised manuscript)

“In reality, some high-quality and low-quality e-sellers adopt the same return policy for similar products. For example, both Estée Lauder and Maybelline have flagship stores on T-mall and offer consumers return insurance. However, some high-quality and low-quality e-sellers adopt different return policies. For instance, on the Taobao platform, the flagship store of The North Face offers consumers return insurance, while the flagship store of Heilan Home does not. These examples suggest that the return insurance policies of e-sellers are not influenced by their competitors.”

**3)** This revision adds cases of Hailan Home and Wancaoyi in the last paragraph of the explanation for Proposition 3 to demonstrate the impact of e-platforms' return insurance policies on e-sellers. Specific changes are as follows: (please refer to the highlighted part in the highlighted section in the first paragraph on page 26 of the revised manuscript)

“In fact, since T-mall began providing consumers monthly return insurance as a gift, some e-sellers have stopped giving return insurance to consumers. For example, the flagship store of China's famous men's wear brand Heilan Home and the outlets store of China's famous women's wear brand Wancaoyi no longer offer return insurance to consumers.”

**(6) Some specific suggestions or opinions can be put forward in the conclusion.**

**The author’s answer:** Dear Reviewer, thank you very much for your suggestions! Based on your advice, I have supplemented the corresponding recommendations in this revision (please refer to the highlighted section in the "Conclusion" part on page 29 of the revised manuscript).

1) Based on the previous two conclusions regarding e-sellers' pricing strategies and return insurance policies, this revision proposes suggestions that focus on the formulation of e-sellers' pricing strategies and return insurance policies.

“Firstly, based on the conditions for e-sellers to offer return insurance, we recommend that e-sellers should comprehensively consider factors such as the product return rate, return compensation, and the commission rate of e-platforms from the perspectives of themselves, insurance companies, and e- platforms to determine the size of the insurance premium before selecting a return strategy. If providing return insurance is not the most suitable return strategy, e-sellers can reduce product prices to attract consumers to purchase by offering discounts and induce consumers to purchase return shipping insurance on their own to reduce return losses.”

2) Based on the final conclusion regarding e-platforms' return insurance policies, this revision proposes suggestions that focus on the e-platforms.

“Secondly, based on the optimal return insurance strategy of e-platforms and its impact on e-sellers, we suggest that e-platforms should play their due role by taking certain measures to increase consumer loyalty without interfering with the market, and should not proactively offer return shipping insurance to consumers.”

Thank you very much for your attention and time. Look forward to hearing from you.

Yours sincerely,

Chen Zhang

5 Aug, 2024

Hainan University

**Supplementary Materials:**

S1 Fig. The Vancl flagship store and the Hailan Home flagship store on T-mall platform (Tif)

S2 Fig. Game sequence diagram (Tif)

S3 Fig. Demand for incomplete substitutes for NN case that the e-platform doesn’t offer return insurance (Tif)

S4 Fig. Demand for incomplete replacement of NO case that the e-platform doesn’t offer return insurance (Tif)

S5 Fig. Demand for incomplete substitutes of ON case that the e-platform doesn’t offer return insurance (Tif)

S6 Fig. Demand for incomplete substitutes of OO case that the e-platform doesn’t offer return insurance (Tif)

S7 Fig. Profit changes of the two e-sellers under different cases when $\frac{h\left(1-\beta\right)\left(1-\alpha_{1}\right)\left(2-h+\alpha_{1}h\right)}{2}<f_{1}<f_{2}$($\alpha_{1}=0.8$, $\alpha_{2}=0.3$, $c_{1}=0.5$, $c_{2}=0.2$, $\beta=0.7$, $s_{1}=0.45$, $s_{2}=0.18$, $r=0.15$, $h=0.15$, $h_{0}=0.001$, $f_{1}=0.03$) (Tif)

S8 Fig. Profit changes of the two e-sellers under different cases when$0<f_{1}<\frac{h\left(1-\beta\right)\left(1-\alpha_{1}\right)\left(2-h+\alpha_{1}h\right)}{2}$($\alpha_{1}=0.8$, $\alpha_{2}=0.3$, $c_{1}=0.5$, $c_{2}=0.2$, $\beta=0.7$, $s_{1}=0.45$, $s_{2}=0.18$, $r=0.15$, $h=0.15$, $h_{0}=0.001$,$f_{1}=0.02$) (Tif)

S9 Fig. Profit changes of e-sellers of different quality under the condition that the e-platform offers return insurance ($\alpha_{1}=0.8$,$\alpha_{2}=0.3$.$c_{1}=0.5$,$c_{2}=0.2$,$\beta=0.7$,$s_{1}=0.45$,$s_{2}=0.18$,$r=0.15$,$h=0.15$,$h_{0}=0.001$,$f_{1}=0.2$) (Tif)

S10 Fig. Profit changes of e-commerce platform before and after providing return insurance ($\alpha_{1}=0.8$,$\alpha_{2}=0.3$.$c_{1}=0.5$,$c_{2}=0.2$,$\beta=0.3$,$s_{1}=0.45$,$s_{2}=0.18$,$r=0.15$,$h=0.15$,$h_{0}=0.001$) (Tif)
