# Supplementary material for: Unlocking the return insurance puzzle in e-commerce: A strategic dance between e-sellers and the e-platform
Source: PLoS One. 2025 May 19;20(5):e0322376. doi: 10.1371/journal.pone.0322376 (PMC12088037; doi:10.1371/journal.pone.0322376)
Supplement: Attachment — Submitted filename: Response_to_Reviewers_auresp_2.docx [file pone.0322376.s004.docx]

Dear Baogui Xin,

Thank you very much for giving us an opportunity to revise our manuscript. We appreciate the editor and reviewers very much for their constructive comments and suggestions on our manuscript entitled ***“Unlocking the Return Insurance Puzzle in E-Commerce: A Strategic Dance Between E-Sellers and E-Platforms”***(PONE-D-24-22624). Those comments are all valuable and very helpful for revising and improving our paper, as well as the important guiding significance to our researches. We have studied comments carefully and have made correction which we hope meet with approval.

First, I would like to explain the situation regarding the delay in submitting the revised manuscript and extend my apologies to the editor.

The revised version has been uploaded to the system. Please kindly check and review it. I sincerely apologize for completing the revisions at this late stage and hope to receive the understanding and acceptance of the editorial office.

The delay occurred because, while working on the manuscript, I received a notification from my school regarding changes to the graduation requirements. As a result, even if this submitted paper were to be accepted, it would not fulfill my graduation requirements, which led me to put it aside for a period of time. However, during my subsequent academic research, I still felt the urge to share this research finding with experts for review and, ideally, have it seen by my peers. Therefore, I have thoroughly revised the paper and responded to each of the expert's comments individually.

I hope the editorial office will grant me this opportunity and take another look at the article. Should there be any issues, please feel free to contact me at any time.

Second, we will reply to the journal requirements.

**Please review your reference list to ensure that it is complete and correct. If you have cited papers that have been retracted, please include the rationale for doing so in the manuscript text, or remove these references and replace them with relevant current references. Any changes to the reference list should be mentioned in the rebuttal letter that accompanies your revised manuscript. If you need to cite a retracted article, indicate the article’s retracted status in the References list and also include a citation and full reference for the retraction notice.**

**The author’s answer:** Dear Editor, thank you for your suggestion! Upon review, the manuscript does not have the aforementioned issues. Thank you once again for your meticulousness.

Third, we will reply to the reviewer’s comments one by one.

**Reviewer #1:**

**After thoroughly reviewing the article, I find that it presents a well-structured and valuable contribution to the field. The research addresses an important and timely issue with a solid theoretical foundation and a well-executed methodology. The discussion is insightful and offers practical implications that can benefit both academics and practitioners.**

**The author’s answer:** Dear Reviewer, I am extremely grateful for your acknowledgment of the revisions made to my manuscript. Once again, thank you for your professional guidance and meticulous suggestions, which have significantly enhanced the structure and content of my manuscript!

**Reviewer #2: 1、Consistency in Terminology: Ensure consistent use of singular or plural forms. For instance, "E-platforms that do offer" should be "E-platform that offer.".**

**The author’s answer:** Dear Reviewer, Thank you for your suggestions and attention to detail! Based on your advice, we have revised the third conclusion in the abstract from "E-platforms that do offer" to "E-platform that offer", highlighted the change in yellow. Additionally, we have carefully read through the entire manuscript and conducted a unified check of all relevant terminology appearing in the text. In response to the inconsistency in terminology you pointed out, we have made individual corrections to ensure consistent use of the same terminology throughout the manuscript.

**2、Unify the line spacing and paragraph format of the entire article, for example, all paragraphs have the first line indented.**

**The author’s answer:** Dear Reviewer, thank you very much for your valuable comment regarding the suggestion to unify the line spacing and paragraph format throughout the entire manuscript! After carefully studying recently published manuscripts in Plos One, we noticed a significant formatting characteristic: the first paragraph following each heading does not use first-line indentation. To align our manuscript 's format with the style of Plos One, we have made uniform adjustments to the line spacing and paragraph format according to the format of recently published manuscripts in Plos One. Except for the first paragraph after each section heading, all other paragraphs maintain a consistent line spacing and adopt first-line indentation.

**3、In "assume ( c2 < c1 < 0.5 )", it could be clearer if this assumption is explicitly tied to the context of the model (e.g., quality of goods). Consider rephrasing for more straightforward interpretation, such as "where c1and c2 represent the unit production costs of higher and lower quality goods, respectively, with c2 < c1 < 0.5 ."**

**The author’s answer:** Dear Reviewer, thank you very much for your valuable suggestion, which we fully agree with! We have added the description "where c1 and c2 represent the unit production costs of higher and lower quality goods, respectively, with c2 < c1 < 0.5" in the last paragraph on page 8 and highlighted it in yellow. The modifications are as follows:

“Unit production cost of goods is $c_{k}$,where $c_{1}$and $c_{2}$ represent the unit production costs of higher and lower quality goods, respectively, with $c_{2}<c_{1}<0.5$.”

**4、The size of images in the article should be consistent, and the fonts in the images should be consistent with the fonts in the article.**

**The author’s answer:** Dear Reviewer, thank you for your valuable suggestion! In response to the issue of inconsistent image sizes and font styles between the images and the text in the article, we have conducted a thorough review of all images and the text contained within them. Subsequently, we have made appropriate adjustments to all images in the manuscript to ensure uniformity in both their sizes and fonts, aligning them seamlessly with the overall style of the manuscript.

**5、Can describe the picture in detail, especially at the intersection.**

**The author’s answer:** Dear Reviewer, thank you for your suggestion! We have further elaborated on the relevant descriptions. For instance, in the first paragraph on page 11, to further describe the image, we have provided the following explanation based on the suggestion "Can describe the picture in detail, especially at the intersection” (highlighted it in yellow):

“As evident from Figs 7 and 8, we find that when the premium of the high-quality e-seller falls within the range of $\left( \frac{h\left( 1-\beta\right)\left( 1-\alpha_{1} \right)\left( 2-h+\alpha_{1}h \right)}{2},f_{2} \right)$, high-quality seller always gain more benefits by not offering return insurance, regardless of whether low-quality seller provide return insurance. Conversely, when the premium is within the range of $\left( 0,\frac{h\left( 1-\beta\right)\left( 1-\alpha_{1} \right)\left( 2-h+\alpha_{1}h \right)}{2} \right)$, offering return insurance is the dominant strategy for high-quality seller, regardless of whether low-quality seller offer return insurance. Therefore, at higher premiums, high-quality e-seller may prefer not to offer return insurance to consumers, and vice versa.”

Final, today is Chinese New Year. Here I wish you a Happy New Year, all the best, prosperity in the Year of the Snake, and joy for your entire family!

Thank you very much for your attention and time. Look forward to hearing from you.

Yours sincerely,

Chen Zhang

28 Jan, 2025

Hainan University
